# Infectious disease ward admission positively influences *P*. *jiroveci* pneumonia (PjP) outcome: A retrospective analysis of 116 HIV-positive and HIV-negative immunocompromised patients

**DOI:** 10.1371/journal.pone.0176881

**Published:** 2017-05-15

**Authors:** Alessandra Ricciardi, Elisa Gentilotti, Luigi Coppola, Gaetano Maffongelli, Carlotta Cerva, Vincenzo Malagnino, Alessia Mari, Ambra Di Veroli, Federica Berrilli, Fabiana Apice, Nicola Toschi, David Di Cave, Saverio Giuseppe Parisi, Massimo Andreoni, Loredana Sarmati

**Affiliations:** 1 Infectious Diseases, Tor Vergata University, Rome, Italy; 2 Respiratory Diseases Unit, Tor Vergata University, Rome, Italy; 3 Department of Haematology, Tor Vergata University, Rome, Italy; 4 Department of Clinical Sciences and Translational Medicine, Tor Vergata University, Rome, Italy; 5 Department of Biomedicine and Prevention, Tor Vergata University, Rome, Italy; 6 Department of Radiology, Athinoula A. Martinos Center for Biomedical Imaging and Harvard Medical School, Boston, MA, United States of America; 7 Department of Molecular Medicine, University of Padua, Padua, Italy, Clinical Microbiology and Virology Unit, Padua University Hospital, Padua, Italy; Kliniken der Stadt Köln gGmbH, GERMANY

## Abstract

*P*. *jiroveci* (*Pj*) causes a potentially fatal pneumonia in immunocompromised patients and the factors associated with a bad outcome are poorly understood. A retrospective analysis on *Pj* pneumonia (PjP) cases occurring in Tor Vergata University Hospital, Italy, during the period 2011–2015. The patients’ demographic, clinical and radiological characteristics and the *Pj* genotypes were considered. The study population included 116 patients, 37.9% of whom had haematological malignancy or underwent haematological stem cell transplantation (HSCT), 22.4% had HIV infection, 16.4% had chronic lung diseases (CLD), 7.8% had a solid cancer, and 3.4% underwent a solid organ transplant (SOT). The remaining 12.1% had a miscellaneous other condition. At univariate analysis, being older than 60 years was significantly correlated with a severe PjP (OR [95%CI] 2.52 [0.10–5.76]; p = 0.031) and death (OR [95%CI] 2.44 [1.05–5.70]; p = 0.036), while a previous trimethoprim/sulfamethoxazole (TMP/SMX) prophylaxis were significantly associated with a less severe pneumonia (OR[95%CI] 0.35 [0.15–0.84], p = 0.023); moreover, death due to PjP was significantly more frequent in patients with CLD (OR[95%CI] 3.26 [1.17–9.05]; p = 0.019) while, admission to the Infectious Diseases Unit was significantly associated with fewer deaths (OR[95%CI] 0.10 [0.03–0.36], p = 0.002). At multivariate analysis, a better PjP outcome was observed in patients taking TMP/SMX prophylaxis and that were admitted to the Infectious Diseases Unit (OR[95%CI] 0.27 [0.07–1.03], p = 0.055, OR[95%CI] 0.16 [0.05–0.55]; p = 0.004, respectively).

In conclusion, in our study population, TMP/SMX prophylaxis and infectious disease specialist approach were variables correlated with a better PjP outcome.

## Introduction

*Pneumocystis jiroveci* pneumonia (PjP) is a serious and potentially life-threatening fungal infection that is mostly diagnosed in immune-compromised individuals. Before the 1980s, it was recognized as a rare, but often fatal, infection that mainly affected patients with haematological malignancies. However, the worldwide epidemic of human immunodeficiency virus (HIV) has dramatically increased the prevalence of PjP, and it has become the most common and alarming complication of the late stages of HIV infection.

Over the last several decades, the introduction of antiretroviral therapy (ARV) dramatically reduced the PjP incidence in HIV-positive patients; however, a new increase in the rate of this pneumonia was reported in large epidemiological studies from France and England [1, 2]. These studies documented a major incidence of PjP in non-HIV-infected patients reaching the levels observed in HIV-positive subjects. The growing number of longer-surviving patients with protracted immunosuppression other than the patients who are traditionally considered in the risk categories is the major cause attributed to this phenomenon. Solid organ transplant (SOT), haematological stem cell transplants (HSCT), chronic lung diseases (CLD) and prolonged treatment with biological drugs (i.e., anti-TNF) or corticosteroids are considered conditions that are associated with an increased risk for PjP development [3]. More efficient laboratory tools for *P*. *jiroveci* (*Pj*) diagnosis and the consequent better ascertainment of PjP cases were also considered the basis for the recent increase in PjP incidence.

Staining methods (May-Grünwald-Giemsa, Gomori methenamine silver, Grocott) were traditionally used to demonstrate Pj in biological specimens, however, despite their good specificity, they failed in detection of low levels of Pj in sputum samples [4, 5]. About twenty years ago, these stains were supplanted by direct or indirect immunofluorescence (IF) using anti—*Pj* monoclonal antibodies: among microscopic methods, IF is considered the most sensitive assay for Pj detection [4]. In recent years, several polymerase chain reaction (PCR) assays directed at various gene targets have been developed to detect *Pj* DNA in respiratory secretions [6–8]. PCR and real time PCR assays are now recommended, in combination of IF, to improve sensitivity and differentiate between PjP and Pj colonization [3, 9]. Moreover, many genetic typing methods and the study of a large number of *Pj* gene loci demonstrated a high level of genetic variability of *Pj* [10–12].

Although these new techniques greatly improve the diagnosis and possibility of early therapy, PjP remains a severe and potentially life-threatening infection. A worse outcome and a higher mortality rate of PjP was reported in HIV-negative patients with respect to patients with HIV-infections [13,14]. Numerous studies have aimed to identify the risk factors associated with a worse outcome of PjP in non-HIV patients and have reported no conclusive results [15–17]. Limited data have been published on the correlation among *Pj* genotypes and clinical outcomes, and the different genotyping systems do not always allow the results obtained by the different studies to be compared [18–20].

The aim of this study was to retrospectively evaluate the clinical and laboratory characteristics related to worse outcomes in 116 PjP cases collected over a five-year period in Tor Vergata University Hospital. In a subgroup of patients, the *Pj* genotypic influence on PjP prognosis was also evaluated.

## Patients and methods

From January 2011 to January 2015, at Tor Vergata University Hospital, Rome, Italy, 507 respiratory samples were collected and evaluated for *Pj* presence.

One hundred thirty-six of the 507 respiratory samples, 98 (72%) bronchoalveolar lavage fluids or bronchoaspirates and 38 (27.9%) sputum samples, were positive for *Pj* (identification of P*j* was based on IF and nested-PCR procedures, see below). A total of 130 patients tested positive, and their medical records were reviewed. One hundred sixteen of these patients received a PjP diagnosis, and 14 were defined as colonized and were thus excluded from the study.

The patients’ demographic characteristics, including the inpatient ward, underlying medical conditions, PjP prophylaxis and treatment, radiological features, severity and outcome of the disease, were harvested by reviewing the patients’ medical records. To collect all of the data of the enrolled patients, a database was created *ad hoc*. In a subgroup of 50 patients of which a sufficient amount of stored samples were available, data on *Pj* genotypes were obtained and considered in a subsequent analysis. No significant differences in terms of sex, age, co-morbidities, diseases severity, radiological findings, co-present lung infections and first-line therapy was found between the subgroup of 50 selected subjects and the rest of population (data not shown)

All of the patients’ personal information was treated in a confidential manner, and all clinical data were collected anonymously to respect the patients’ privacy. The study was approved by the Tor Vergata University Ethical Committee.

## Definitions

### PjP

PjP was defined by clinical signs of pneumonia (cough, dyspnoea and fever) and by pathological changes of radiological examination (bilateral reticular or granular infiltrates, focal consolidation, unilateral infiltrates, or nodules) associated with the simultaneous presence of *Pj* in respiratory secretions.

In the respiratory secretions of 32 patients, *Pj* was present with other pathogens. These cases were considered to be the result of concomitant infections, and patients received therapy for both pathogens. These cases were defined in the text as co-present lung infections.

At admission, PjP was considered severe when the patient had a Pa0_2_ <70 mm Hg in room air and required supplemental oxygen, non-invasive mechanical ventilation or endotracheal intubation.

A positive outcome of PjP was defined as clinical improvement and survival after 90 days from the pneumonia diagnosis.

### Pj colonization

*Pj* colonization, in contrast to *Pj* infection, was defined by the positive detection of *Pj* DNA by PCR detection without signs or symptoms of acute pneumonia.

### Typical and atypical high-resolution computed tomography (CT) PjP radiological findings

According to previous published data [21], a fine reticular interstitial pulmonary pattern with a prevalent perihilar distribution as well as ground glass opacities, small pneumatocoeles and/or sub-pleural blebs were considered typical PjP radiological findings. Dense lobar consolidation, nodules, miliary opacities, and pleural effusions are uncommon radiological aspects of PjP and were thus defined as ‘atypical radiological findings’.

## *Pj* laboratory diagnosis

### Pj detection and genotyping

For all samples, the detection of P*j* was based on immunofluorescent procedures with monoclonal antibodies (MERIFLUOR® Pneumocystis, Meridian Diagnostic, Cincinnati, OH, USA) and nested-PCR to amplify 260 bp of the *mtLSU-rRNA* region as described [22]. Genomic DNA was extracted from biological samples using the QIAamp DNA kit (QIAGEN, Hilden, Germany) according to the manufacturer’s instructions. All PCRs were carried out in 25 μL volumes containing 12.5 μL of PCR master mix 2X (Promega Italia, Milan, Italy), 5 μl of template DNA (1 μl of the primary PCR products in the second round of PCR), and 0.6 mM of each primer, under the following conditions: 2 min at 95°C, followed by 35 cycles of 1 min at 95°C, 1 min at 55°C, 1 min at 72°C, and a final elongation of 7 min at 72°C. In all PCR reactions, positive and negative controls were added. Amplicons were visualized by electrophoresis on SYBR Safe DNA-stained 1% agarose gels (Invitrogen, Life Technologies, Monza, Italy).

A total of 50 samples (DNA and/or amplicons) still available from existing collections were used for genotyping by sequencing. DNA was purified using the mi-PCR Purification Kit (Metabion International AG, Planneg, Germany) and sequenced in both directions by the Bio-Fab Research (Rome, Italy). Consensus sequences were edited using the FinchTV 1.4 software (Geospiza, Inc, Seattle, WA, USA), queried against known *Pj* genotypes retrieved from GenBank and then used for identification.

## Statistical analysis

A descriptive analysis of categorical variables was performed with frequency tables, while for continuous variables, medians, ranges and standard deviations were computed. Chi squared tests, Fischer’s tests and a log binomial regression model were adopted for both univariate and multivariate analyses to detect the association between predictor variables and PjP severity and outcomes. Covariates with a p-value ≤0.05 were considered for multivariate analysis. Statistical significance was assessed for p-values ≤0.05. Statistical analysis was performed using the IBM SPSS software version 21, NY, U.S.A.

## Results

The clinical characteristics of the 116 patients with a diagnosis of PjP are shown in Table 1. Most (60.3%) of the patients were male, 40% of whom were over sixty. Forty percent of all PjP cases were admitted to the Infectious Diseases Unit. Onco-haematologic diseases and HSCT were the most common underlying diseases (37.9%), followed by HIV infection (22.4%), CLD (16.4%), solid cancer (7.8%) and SOT (3.4%). In the group defined as ‘other clinical conditions’, miscellaneous other conditions (inflammatory bowel diseases, rheumatologic diseases, anorexia or a previous major surgery) associated with immunosuppression were recorded. This group of patients represented 12% of the entire population.

**Table 1:** General characteristics of 116 patients affected by PjP, patients’ number and percentage (%).

Characteristics	
**N. of patients**	116
**Sex**	**N. (%)**
Male	70 (60.3)
**Age**	
Mean, years, range, SD	54.4, 20–87 (16.65)
Age > 60 yrs (%)	47 (40.5)
**Ward of admission**	**N. (%)**
Infectious diseases	46 (39.7)
Haematology/HSCT	36 (31.0)
Respiratory diseases	20 (17.2)
Other	14 (12.1)
**Underlying diseases**	**N. (%)**
HIV	26 (22.4)
Haematological malignancy /HSCT	44 (37.9)
Solid cancer	9 (7.8)
CLD	19 (16.4)
SOT	4 (3.4)
Other clinical conditions	14 (12.1)
**Co-present lung infections (tot 32)**	**N. (%)**
Aspergillosis	11 (34.4)
Pneumococcal pneumonia	5 (15.6)
Other bacterial pneumonia	10 (31.2)
CMV pneumonia	2 (6.3)
Tuberculosis	4 (12.5)
**Lung TC scan findings**	**N. (%)**
Typical	83 (71.6)
Atypical	33 (28.4)
**Genotype (tot 50)**	**N. (%)**
1	22 (44.0)
2	10 (20.0)
3	15 (30.0)
1/2	1 (2.0)
1/3	1 (2.0)
2/3	1 (2.0)
**Previous PjP prophylaxis**	**N (%)**
Yes	28 (24.1)
No	87 (75.0)
Not known	1 (0.9)
**Disease severity**	**N (%)**
Severe	75 (64.7)
Not severe	41 (35.3)
**Clinical course**	**N (%)**
Recovery	86 (74.1)
Death	30 (25.9)
**First line therapy**	**N (%)**
TMP/SMX	90 (77.6)
Caspofungin	10 (8.6)
TMP/SMX+Caspofungin	6 (5.2)
Caspofungin+Atovaquone+Primaquine+Pentamidine	2 (1.7)
Other	2 (1.7)
Not known	6 (5.2)

PjP = Pneumocystis jiroveci pneumonia; SD = standard deviation; HSCT = Haematological Stem cell transplantation; HIV = Human Immunodeficiency Virus; CLD = Chronic Lung Diseases; SOT = Solid Organ Transplant; CMV Cytomegalovirus; TC = computed tomography;TMP/SMX = trimethoprim/sulfamethoxazole

In 32 (27.5%) patients, *Pj* was present in concomitance with other lung infections: in most cases (34.4%) with pulmonary aspergillosis, in 4 cases (12.5%) with *Mycobacterium tuberculosis*, in 2 cases with Cytomegalovirus and in the remaining 15 cases with bacterial infections, one-third (5 patients) of which were due to *Streptococcus pneumoniae*. Five (15.6%) of these patients were HIV-positive (data not shown).

Typical PjP radiological findings were present in 71.6% of the cases. Sixty-five percent of the patient population had a severe PjP, and 26% of the study population died due to respiratory failure.

Only 24.1% of the studied subjects were on trimethoprim/sulfamethoxazole (TMP/SMX) prophylaxis before the PjP occurrence, and the large majority of PjP received TMP/SMX treatment. The median duration of PjP therapy was 21 days.

The genotypic analysis of *Pj* in the subgroup of 50 patients demonstrated that 44% of the patients had genotype 1, 30% had genotype 3 and 10% had genotype 2. In 3 cases (6%), a mixed genotype infection was found.

The results of a univariate analysis, which aimed to identify the variables related to PjP severity and outcomes, are reported in Table 2.

**Table 2:** Univariate analysis of risk factors associate with severe pneumonia and death in 116 patients with PjP.

	Risk factors associate with severe PjP					Risk factors associate with death due to PjP				
	Severe N (%)	Not Severe N (%)	OR	95% CI	*p*-value	Death N (%)	Resolution N (%)	OR	95% CI	*p*-value
**Underlying diseases**					0.071					**0.017**
HIV	20 (76.9)	6 (23.1)	3.33	1.12–9.88		3 (11.5)	23 (88.5)	0.59	0.14–2.44	
Haematol. dis./HSCT	22 (50.0)	22 (50.0)	1.00			8 (18.2)	36 (81.8)	1.00		
Solid Cancer	7 (77.8)	2 (22.2)	3.50	0.65–18.76		4 (44.4)	5 (55.6)	3.60	0.79–16.49	
CLD	13 (68.4)	6 (31.6)	2.18	0.70–6.73		9 (47.4)	10 (52.6)	4.05	1.24–13.21	
SOT	4 (100)	0 (0)	**-**	-		0 (0)	4 (100)	**-**	-	
Other	9 (64.3)	5 (35.7)	1.80	0.52–6.24		6 (42.9)	8 (57.1)	3.38	0.91–12.45	
**CLD vs other**					0.708					**0.019**
CLD	13 (68.4)	6 (31.6)	1.22	0.43–3.50		9 (47.4)	10 (52.6)	3.26	1.17–9.05	
Other diseases	62 (63.9)	35 (36.1)	1.00			21 (21.6)	76 (78.4)	1.00		
**Age cut off 60 y**					**0.031**					**0.036**
≤60	39 (56.5)	30 (43.5)	1.00			13 (18.8)	56 (81.2)	1.00		
>60	36 (76.6)	11 (23.4)	2.52	0.10–5.75		17 (36.2)	30 (63.8)	2.44	1.05–5.70	
**TMP/SMX PPX**					**0.023**					0.254
Yes	13 (46.4)	15 (53.6)	0.35	0.15–0.84		5 (17.9)	23 (82.1)	0.54	0.18–1.58	
No	62 (71.3)	25 (28.7)	1.00			25 (28.7)	62 (71.3)	1.00		
**TC result**					**0.009**					0.247
Typical	60 (72.3)	23 (27.7)	1.00			19 (22.9)	64 (77.1)	1.00		
Atypical	15 (45.5)	18 (54.5)	0.32	0.14–0.74		11 (33.3)	22 (66.7)	1.68	0.69–4.09	
**First-line therapy**					0.068					0.599
TMP/SMX	55 (61.1)	35 (38.9)	1.00			18 (20.0)	72 (80.0)	1.00		
Caspofungine	8 (80.0)	2 (20.0)	0.55	0.51–12.69		3 (30.0)	7 (70.0)	1.71	0.40–7.29	
TMP/SMX + CAS	6 (100)	0 (0.0)	-	**-**		2 (33.3)	4 (66.7)	2.00	0.34–11.79	
CAS+PEN/PR/CLIN/ATO	2 (100)	0 (0.0)	-	**-**		1 (50.0)	1 (50.0)	4.00	0.24–67.1	
Other	1 (50.0)	1 (50.0)	0.63	0.04–10.51		1 (50.0)	1 (50.0)	4.00	0.24–67.1	
**Co-present lung inf.**					0.893					0.731
Yes	21 (34.4)	11 (65.6)	1.06	0.45–2.49		9 (28.1)	23 (71.9)	1.17		
No	54 (64.3)	30 (35.7)	1.00			21 (25.0)	63 (75.0)	1.00	0.47–2.93	
**Ward of admission**					**0.027**					**0.002**
Infectious Dis.	32 (69.6)	14 (30.4)	1.00			5 (10.9)	41 (89.1)	0.10	0.03–0.36	
Haematology/HSCT	17 (47.2)	19 (52.8)	0.39	0.16–0.97		10 (27.8)	26 (72.2)	0.32	0.10–0.99	
Respiratory Dis	17 (85.0)	3 (15.0)	2.48	0.62–9.84		11 (55.0)	9 (45.0)	1.00		
Other	9 (64.3)	5 (35.7)	0.79	0.22–2.79		4 (28.6)	10 (71.4)	0.33	0.07–1.40	
**Respiratory Dis. vs others Wards**					**0.036**					
Respiratory Dis	17 (85.0)	3 (15.0)	3.71	1.02–13.5						
Other wards	58 (60.4)	38 (39.6)	1.00							
**Infectious Dis. vs others Wards**										**0.002**
Infectious Dis.						5 (10.9)	41 (89.1)	1.00		
Other wards						25 (35.7)	45 (64.3)	4.56	1.60–13.0	

PjP = Pneumocystis jiroveci pneumonia; OR = odds ratio, CI = confidence interval, significance at α = 0.05;; HIV = Human Immunodeficiency Virus; Haematol. Dis. = Haematological diseases; HSCT = Haematological Stem cell transplantation; CLD = Chronic Lung Diseases; SOT = Solid Organ Transplant; TMP/SMX = trimethoprim/sulfamethoxazole; PPX = prophylaxis;; TC = computed tomography; CAS = caspofungin; CAS+PEN/PR/CLIN/ATO = Caspofungin+Atovaquone/ Primaquine/Clindamicine/Atovaquone

Being older than 60 years was significantly correlated with a severe PjP (OR [95%CI] 2.52 [0.10–5.76]; p = 0.031), and a previous TMP/SMX prophylaxis and an atypical PjP radiological finding were significantly associated with a less severe pneumonia (OR[95%CI] 0.35 [0.15–0.84], p = 0.023, OR[95%CI] 0.32 [0.14–0.74], p = 0.009, respectively). A significant difference in the number of patients with a severe PjP admitted in the different wards was demonstrated (p = 0.027); in particular, a significantly higher proportion of severe PjP patients was admitted to the Respiratory Diseases Unit (OR[95%CI] 3. 71 [1.02–13.5], p = 0.036).

The variables that were significantly associated with the worst PjP outcome and death were being older than 60 years (OR[95%CI] 2.44 [1.05–5.70]; p = 0.036) and suffering from a CLD (OR[95%CI] 3.26 [1.17–9.05]; p = 0.019). In contrast, admission to the Infectious Diseases ward was a protective factor against a bad outcome and was significantly associated with fewer deaths (OR[95%CI] 0.10 [0.03–0.36], p = 0.002).

In Table 3 the main differences between patients admitted to Infectious Diseases ward versus those admitted to the other units were reported

**Table 3:** General characteristics of patients with PjP admitted to Infectious Diseases ward versus those admitted on other wards.

	Infectious Diseases N (%)	Other wards N(%)	OR	95% CI	P value
**Sex, N (%)**					0.102
Male	32 (45.7)	38 (54.3)	1.00	-	
Female	14 (30.4)	32 (69.6)	1.93	0.88–4.22	
**Age**					0.527
Age ≤ 60 yrs (%)	29 (42.0)	40 (58.0)	1.00	-	
Age > 60 yrs (%)	17 (36.2)	30 (63.8)	0.78	0.36–1.68	
**Underlying diseases N (%)**					**0.000**
HIV	21 (80.8)	5 (19.2)	0.07	0.02–0.23	
Hematological malignancy /HSCT	10 (22.7)	34 (77.3)	1.00	-	
Solid cancer	4 (44.4)	5 (55.6)	0.37	0.08–1.63	
CLD	2 (10.5)	17 (89.5)	2.50	0.49–12.7	
Solid organ transplantation	2 (50.0)	2 (50.0)	0.29	0.04–2.36	
Miscellaneous	7 (50.0)	7 (50.0)	0.29	0.08–1.04	
**Lung TC scan findings N (%)**					0.920
Typical**	36 (43.4)	47 (56.6)	1.00	-	
Not typical	8 (42.1)	11 (57.9)	1.05	0.38–2.89	
**Type of respiratory sample N (%)**					0.267
BAL/ Broncho aspirate	30 (38.5)	48 (61.5)	0.63	0.27–1.43	
Sputum	16 (50.0)	16 (50.0)	1.00	-	
**Co-present lung infections N (%)**					0.555
Yes	7 (46.7)	8 (53.3)	0.72	0.24–2.14	
No	39 (38.6)	62 (61.4)	1.00	-	
**Genotype (tot 50) N (%)**					**0.000**
1	6 (27.3)	16 (72.7)	1.00	-	
2	9 (90.0)	1 (10.0)	0.04	0.00–0.40	
3	11 (73.3)	4 (26.7)	1.36	0.03–0.60	
mixed	3 (100)	0	-	-	
**Previous PCP prophylaxis N (%)**					**0.000**
Yes	3 (10.7)	25 (89.3)	8.14	2.29–28.9	
No	43 (49.4)	44 (50.6)	1.00	-	
**Disease severity**					0.368
Severe	32 (42.7)	43 (57.3)	1.00	-	
Not severe	14 (34.1)	27 (65.9)	1.44	0.65–3.12	
**First line therapy**					0.985
TMP/SMX	36 (40.0)	54 (60.0)	1.00	-	
Caspofungin	4 (40.0)	6 (60.0)	1.00	0.26–3.80	
TMP/SMX+Caspofungin	3 (50.0)	3 (50.0)	0.68	0.13–3.49	
Caspofungin+Atovaquone+Primaquine+Pentamidine	1 (50.0)	1 (50.0)	0.68	0.04–11.0	
Other	1 (50.0)	1 (50.0)	0.68	0.04–11.0	
**Clinical course**					**0.002**
Recovery	41 (47.7)	45 (52.3)	1.00	-	
Death	5 (16.7)	25 (83.3)	0.22	0.08–0.63	

PjP = Pneumocystis jiroveci pneumonia; OR = odds ratio, CI = confidence interval, significance at α = 0.05;; HIV = Human Immunodeficiency Virus; Haematol. Dis. = Haematological diseases; HSCT = Haematological Stem cell transplantation; CLD = Chronic Lung Diseases; SOT = Solid Organ Transplant; TMP/SMX = trimethoprim/sulfamethoxazole; PPX = prophylaxis;; TC = computed tomography; BAL = bronchoalveolar lavage; CAS = caspofungin; CAS+PEN/PR/CLIN/ATO = Caspofungin+Atovaquone/ primaquine/Clindamicine/Atovaquone

Of the 46 patients admitted to the Infectious Disease ward, the majority (25, 54%) were HIV-negative; of the latter, 10 had a haematological malignancy or HSCT, 4 had solid tumours, 2 had CLD, 2 were SOT, and 7 had one of the so-called other clinical conditions.

With respect to other wards, a significant lesser number of patients with CLD and a significant higher number of patients with Pj genotype 3 were admitted in Infectious Diseases Unit (OR [95%CI] 2.50 [0.49–12.7], p<0.0001; OR [95%CI] 1.36 [0.03–0.60], p<0.0001, respectively). A significant lesser number of patients on TMP/SMX prophylaxis were hospitalized in Infectious Diseases ward, nevertheless in this ward there were significantly fewer deaths (OR [95%CI] 8.14 [2.29–28.9], p<0.0001 OR [95%CI] 0.22 [0.08–0.63], p<0.002, respectively).

Multivariate analysis (Table 4) showed that a better outcome were observed in those patients who were admitted to the Infectious Diseases ward (OR [95%CI] 0.16 [0.05–0.55]; p = 0.004). A correlation, very close to statistical significance, with a better outcome was also demonstrated for the previous use of TMP/SMX prophylaxis (OR[95%CI] 0.27 [0.07–1.03], p = 0.055).

**Table 4:** Multivariate analysis of predictors of PjP severity and death in 116 patients with PjP.

	Multivariate analysis severity		Multivariate analysis death	
	OR (95% CI)	*p*-value	OR (95% CI)	*p*-value
**CLD vs other pathologies**	0.63 (0.18–2.20)	0.464	1.45 (0.43–4.87)	0.544
**Age >60 yrs vs ≤60 yrs**	1.93 (0.79–4.72)	0.152	2.53 (0.94–6.82)	0.068
**TC result** **not typical vs typical**	0.42 (0.17–1.03)	0.059	2.97 (0.99–8.95)	**0.053**
**Co-present lung infections** **(yes vs no)**	1.07 (0.43–2.68)	0.878	1.55 (0.54–4.39)	0.413
**Infectious Diseases Wards** **vs Other Wards**	0.91 (0.34–2.42)	0.844	0.16 (0.05–0.55)	**0.004**
**Prophylaxis administered** **(yes vs no)**	0.39 (0.13–1.12)	0.079	0.27 (0.07–1.03)	**0.055**

OR = odds ratio, CI = confidence interval, significance at α = 0.05 PjP = Pneumocystis jiroveci pneumonia; CLD = Chronic Lung Diseases; TC = computed tomography

Being older than 60 years and having typical PjP CT findings were associated with a worse outcome and death (OR[95%CI] 2.53 [0.94–6.82], p = 0.068 and OR[95%CI] 2.97 [0.99–8.95], p = 0.053, respectively).

The correlations among *Pj* genotypes, clinical PjP characteristics and outcomes of the subgroup of 50 patients are reported in Table 5.

**Table 5:** Genotype characteristics of 50 patients affected by PjP.

	Overall (tot 50)	Gen 1 (tot 22)	Gen 2 (tot 10)	Gen 3 (tot 15)	Gen mixed (tot 3)	*p*-value
**Sex, N (%)**						**0.290**
Male	30 (60.0)	11 (50.0)	8 (80.0)	10 (66.7)	1 (33.3)	
Female	20 (40.0)	11 (50.0)	2 (20.0)	5 (33.3)	2 (66.7)	
OR (95% CI)		1.00	0.25 (0.04–1.45)	0.50 (0.13–1.95)	2.00 (0.16–25.4)	
**Age**						**0.242**
≤ 60 yrs (%)	28 (56.0)	10 (45.5)	5 (50.0)	10 (66.7)	3 (100.0)	
> 60 yrs (%)	22 (44.0)	12 (54.5)	5 (50.0)	5 (33.3)	0	
OR (95% CI)		1.00	0.83 (0.19–3.72)	0.42 (0.11–1.63)	-	
**Underlying diseases N (%)**						**0.214**
HIV	16 (32.0)	6 (27.3)	3 (30.0)	4 (26.7)	3 (100.0)	
Other	34 (68.0)	16 (72.7)	7 (70.0)	11 (73.3)	0	
OR (95% CI)		1.00	0.87 (0.17–4.54)	1.03 (0.24–4.53)	-	
**TC findings (tot 48) N (%)**						**0.434**
Typical	39 (78.0)	16 (84.2)	9 (100.0)	11 (78.6)	3 (100.0)	
Not typical	11 (22.0)	3 (15.8)	0	3 (21.4)	0	
OR (95% CI)		1.00	-	1.45 (0.25–8.58)	-	
**Respiratory sample N (%)**						**0.394**
BAL/ Broncho-aspirate	34 (68.0)	17 (77.3)	7 (70.0)	9 (60.0)	1 (33.3)	
Sputum	16 (32.0)	5 (22.7)	3 (30.0)	6 (40.0)	2 (66.7)	
OR (95% CI)		1.00	0.69 (0.13–3.68)	0.44 (0.11–1.85)	0.15 (0.01–1.98)	
**Co-present infections N(%)**						**0.243**
Yes	11 (22.0)	5 (22.7)	0	5 (33.3)	1 (33.3)	
No	39 (78.0)	17 (77.3)	10 (100.0)	10 (66.7)	2 (66.7)	
OR (95% CI)		1.00	-	1.70 (0.39–7.36)	1.70 (0.13–22.9)	
**PjP PPX N (%)**						**0.675**
Yes	6 (12.0)	2 (9.1)	1 (10.0)	2 (13.3)	1 (33.3)	
No	44 (88.0)	20 (90.9)	9 (90.0)	13 (86.7)	2 (66.7)	
OR (95% CI)		1.00	1.11 (0.09–13.9)	1.54 (0.19–12.3)	5.00 (0.30–82.7)	
**Disease severity**						**0.738**
Severe	39 (78.0)	17 (77.3)	7 (70.0)	12 (80.0)	3 (100.0)	
Not severe	11 (22.0)	5 (22.7)	3 (30.0)	3 (20.0)	0	
OR (95% CI)		1.00	0.68 (0.13–3.68)	1.18 (0.24–5.90)		
**Clinical course (tot 51) No (%)**						**0.398**
Recovery	39 (78.0)	15 (68.2)	9 (90.0)	12 (80.0)	3 (100.0)	
Death	11 (22.0)	7 (31.8)	1 (10.0)	3 (20.0)	0	
OR (95% CI)		1.00	4.20 (0.44–39.9)	1.87 (0.40–8.80	-	

PjP = Pneumocystis jiroveci pneumonia; OR = odds ratio, CI = confidence interval, significance at α = 0.05;; HIV = Human Immunodeficiency Virus; PPX = prophylaxis;; TC = computed tomography; BAL = bronchoalveolar lavage

No correlation was found between the 3 genotypes and any of the demographic or clinical characteristics that were evaluated. A trend for a lesser association between genotype 2 and a co-present infection was found. All 3 subjects with mixed *Pj* genotypes in respiratory secretions had HIV infections and a severe PjP with a typical TC scan finding. Patients with haematological illness were more frequently infected by genotypes 2 and 3, patients with CLD by genotype 1, and HIV-positive patients by all 3 genotypes. No correlation was found between PjP severity and genotype. Most deaths were associated with *Pj* genotype 1 infection, but the datum was not significant.

## Discussion

In this retrospective study conducted on a cohort of HIV-positive and HIV-negative patients with PjP, in the univariate analysis, an age >60 years and CLD were significantly associated with more severe outcomes and death due to respiratory failure, whereas receiving TMP/SMX prophylaxis was associated with a reduced possibility of severe PjP. The multivariate analysis, indicated that significantly fewer deaths due to PjP were recorded in the Infectious Diseases ward, although more severe PjP patients were admitted to this ward.

Age is a predictor that is widely used in risk stratification rules for community-acquired pneumonia, and older age has been associated with a worse PjP prognosis in both HIV-positive and HIV-negative subjects [23–26]. The correlation between older age and death due to PjP was recently confirmed by Roux A. and colleagues [27] in a large prospective cohort study, in which 544 PjP cases were collected and analysed in France. This study demonstrated an increased risk of death due to PjP for each additional year over 50 years of age.

At univariate analysis, a higher number of deaths was reported for patients with CLD who were admitted to the Respiratory Unit. It has been demonstrated that *Pj* colonization worsens bronchoconstriction in patients with chronic obstructive pulmonary disease by stimulating local inflammation process so damaging lung function[28]. This phenomenon could facilitate respiratory failure of patients with CLD and in part explain the significant higher number of deaths from PjP in this group patients. However, patients with CLD are not generally considered a high-risk category for PjP, so it is also possible that there was a delay in diagnosis in this group of patients.

Our results showed a benefit of *Pj* prophylaxis on PjP severity. The effect of previous TMP/SMX prophylaxis on the severity of PjP is controversial. Previously published articles on HIV-positive patients [29,30] have demonstrated that the prior receipt of TMP/SMX was associated with a poor outcome from severe PjP, and this phenomenon was ascribed to possible TMP/SMX resistance acquisition. However, a correlation between the lack of prophylaxis and a worse PjP outcome in non-AIDS patients was recently reported in a study from France [27], and a Cochrane analysis [31] clearly showed the efficacy of *Pj* prophylaxis among non-HIV immunocompromised patients. An appropriate use of prophylaxis does not exclude its possible failure, even if it is possible to argue that the amount of *Pj* in the lungs of patients under prophylaxis is lower, thus inducing a less severe PjP clinical picture. On the contrary, the physician’s risk perception of PjP in patients who are not taking prophylaxis because they are not considered to be at risk, may be poor, and this could delay the diagnostic process and subsequent treatment, thereby worsening the prognosis. In our study, which included numerous categories of immune compromised hosts, only 24% of the patients had taken TMP/SMX prophylaxis before pneumonia onset. Thus, it is possible that the risk of PjP has been underestimated. In our opinion, it is necessary to reconsider the groups of patients who are at risk for PjP and could benefit from prophylaxis and to ensure good compliance with prophylaxis by those patients for whom the use of TMP/SMX has been established by the currently used guidelines.

In the multivariate model, age did not show to be statistically related to the PjP severity or death. This could be related to the sample size and to the fact that age could be influenced by other variables, such as the underlying pathology. As for the TMP/SMX prophylaxis, results show that there is a trend through the significance, being the p-value close to the cut off we considered for the significance. Surprisingly, in both univariate and the multivariate analysis of our study population, although the number of severe PjP cases was high, admission to Infectious Disease ward was associated with a significantly better outcome and fewer deaths. A significantly lower number of patients with CLD was admitted to Infectious Diseases ward and this could justify the better PjP outcome in this unit. However, an almost double number (32 versus 17) of serious PjP were admitted to Infectious Diseases respect to Respiratory Diseases ward, in addition, at multivariate analysis the condition of CLD did not correlate either with the clinical severity nor with death by PjP. A comparable number of HIV-positive and HIV-negative patients were hospitalized in the Infectious Diseases ward during the study period, and no significant differences in the outcomes of the two groups of patients were detected.

To our knowledge, this is the first report in which the infectious disease specialist approach was related to a better prognosis of PjP. However, other authors have suggested that the difference between being HIV-positive and HIV-negative in PjP prognosis is secondary to a limited physicians’ alertness for HIV-negative patients regarding PjP diagnosis with a consequent delay in the beginning of an appropriate treatment [24]. The HIV-infected patients were predominantly treated by infectious disease specialists, whereas most of the non-HIV-infected patients were usually under the charge of medical professionals other than infectious disease specialists. The incidence of PjP in immunocompromised patients without HIV is increasing [32], and chemotherapy, HSCT and solid organ transplant, and corticosteroids or other immune suppressive medications are all considered risk factors for PjP development. However, with the exception of HIV infection, the general perception of PjP risk in other categories of immunosuppression is low, and a recent report from the Fifth European Conference on Infections in Leukemia (ECIL-5) stated that patients with acute lymphoblastic leukaemia or HSCT recipients who develop PjP usually do not receive adequate prophylaxis [33].

Previous reports showed that hospitalized patients with severe infections are significantly less likely to die if they receive care from an infectious diseases specialist and also that the benefits of infectious diseases specialist consultation are more pronounced when patients are seen earlier [34, 35]. All of these considerations led to the conclusions that the risk of PjP can be underestimated in immunosuppressed HIV-negative patients, usually not managed by the infectious disease specialist, and that a reduced risk perception in the patient assessment delays the diagnostic process. An early diagnosis is critical for optimal management and for the rapid introduction of a proper treatment, thereby influencing the outcome [23, 26].

One of the aims of our study was to evaluate the possible correlation between *Pj* genotypes and outcomes; however, the reduced number of patients who were studied limited the analysis, which resulted in no significant results.

Before we can discuss the conclusions, some of the limitations of our study need to be discussed. The retrospective nature of the study was its major limitation. The limited number of cases collected in the different groups of underlying diseases did not allow us to detect any prognostic differences among the different types of immunosuppression. Moreover, we could not evaluate the influence of different treatments, and we collected no data on the time elapsed between admission and the initiation of treatment, which recent publications have reported as being correlated with PjP prognoses [23]. Furthermore, all patients were from a single institution, which might reduce the generalizability of the conclusions.

In conclusion, a specialist approach and TMP/SMX prophylaxis were all variables associate with a better PjP outcome in immunocompromised hosts in our study population. More patients need to be studied to understand whether *Pj* genotype characterization could be useful in the prognostic evaluation of patients with PjP.
